# Utilization of insecticide-treated bed nets and key factors among households in resource-limited settings, Northwest Ethiopia

**DOI:** 10.1186/s12936-025-05698-8

**Published:** 2025-11-28

**Authors:** Abraham Teym, Tirsit Ketsela Zeleke, Mestet Yibeltal Shiferaw, Gete Berihun, Mengistu Abebe Messelu, Temesgen Aynew, Ayenew Sisay Gebeyew, Bayou Tilahun Assaye, Almaw Genet Yeshiwas, Chalachew Yenew

**Affiliations:** 1 Department of Environmental Health, College of Health Sciences, Debre Markos University, Debre Markos, Ethiopia; 2 Department of Pharmacy, College of Health Sciences, Debre Markos University, Debre Markos, Ethiopia; 3 Department of Surgery, Neurosurgery Unit, Injibara University, Injibara, Ethiopia; 4 Department of Nursing, College of Medicine and Health Sciences, Debre Markos University, Debre Markos, Ethiopia; 5 Department of Health Informatics, College of Medicine and Health Sciences, Debre Markos University, Debre Markos, Ethiopia; 6 Department of Environmental Health, College of Medicine and Health Sciences, Injibara University, Injbara, Ethiopia; 7 Department of Public Health, College of Medicine and Health Sciences, Debre Tabor University, Debre Tabor, Ethiopia

**Keywords:** Households, Insecticide treated bed nets, Key factors, Utilization, Malaria prevention, District, Ethiopia

## Abstract

**Background:**

Insecticide-treated mosquito nets (ITNs) act as an essential tool in malaria prevention by serving as a physical barrier against the disease. In sub-Saharan Africa, the use of ITNs remains a cornerstone in efforts to reduce malaria transmission. Despite their importance, there is limited evidence on ITN utilization and the factors influencing their use in Ethiopia.

The objective of this study was to assess utilization of insecticidal treated bed nets and its key factors among Households **i**n resource-limited settings, Northwest, Ethiopia, in 2024.

**Methods:**

A community-based cross-sectional study was carried out from January 30 to March, 2024. A total of 492 randomly selected Households were included. Data were acquired using a pre-tested structured questionnaire and an observational checklist. Binary logistic regression was initially used to select variables associated with the use of insecticide-treated bed nets. Variables with a p-value less than 0.25 in the bivariable analysis were selected for inclusion in the multivariable logistic regression model. In the multivariable analysis, factors with a p-value less than 0.05 and 95% confidence intervals were considered statistically significant.

**Results:**

A total of 492 study participants responded to the questionnaire, resulting in a response rate of 98.4%. Approximately 71.3% (95% CI 67.2%, 74.9%) of the respondents reported utilizing insecticide-treated nets on the night before the data collection day. Factors significantly associated with ITN utilization included being female (AOR = 1.538; 95% CI 1.157, 3.135), households with illiterate educational status (AOR = 0.237; 95% CI 0.026, 0.871), households with monthly income > 1000 ETB (AOR = 1.372; 95% CI 1.038, 3.184), and households that had received information (messages) about ITN use (AOR = 1.310; 95% CI 1.125, 3.229).

**Conclusion:**

The utilization of insecticide-treated bed nets (ITNs) among households was 71.3%, which was lower than the WHO recommendation (80%). Key factors significantly associated with ITN utilization included being female, educational status, higher household income, and receiving information (messages) about ITN use. Targeted interventions, such as community education and awareness campaigns, are essential to improve ITN utilization. Addressing these factors can close the gap with WHO standards and reduce the malaria burden effectively.

**Supplementary Information:**

The online version contains supplementary material available at 10.1186/s12936-025-05698-8.

## Background

Insecticide-treated nets (ITNs) are designed to act as both a physical and chemical barrier against mosquitoes, offering a cost-effective approach to malaria prevention [1]. To maintain their efficacy, ITNs must be treated with insecticide regularly or replaced when necessary. Their use is particularly crucial in regions with high malaria transmission, as they are one of the most effective tools for controlling the disease [2–4]. Malaria remains a major public health problem, with almost half of the world's population at risk [5].

The World Health Organization (WHO) reported in 2020 that malaria remains a significant global health challenge, with an estimated 229 million cases recorded across 87 endemic countries. Tragically, nearly 409,000 deaths were attributed to malaria that year, the majority of which 67% occurred in children under five years old [6]. The WHO African Region bore the highest burden, accounting for approximately 94% of these cases, equating to 215 million infections [7, 8]. Malaria imposes a substantial burden on healthcare systems, particularly in sub-Saharan Africa. It is responsible for a significant proportion of healthcare utilization, contributing to 25–35% of outpatient consultations, 20–45% of hospital admissions, and 15–35% of hospital-related deaths [9]. This disease continues to strain the already limited healthcare resources in the region [10].

Malaria causes millions of cases and hundreds of deaths in Ethiopia every year [11]. Malaria is a significant public health issue in Ethiopia [12]. Annually, up to 6 million clinical cases have been recorded. However, the estimated number of clinical malaria cases ranges from 8 to 10 million [13]. Malaria has been cited as the leading cause of illness and mortality. Malaria accounts for 16.6% of outpatient visits, 15% of hospitalisations, and 28.9% of inpatient mortality [13]. Approximately three-quarters of Ethiopia's total territory is malarial, and an estimated 48 million people, 68% of the population, reside in malaria-risk areas [14, 15]. Regardless of the fact that numerous malaria prevention mechanisms exist, the WHO suggested the use of insecticide-treated bed nets (ITNs) as one of the measures to reduce the deleterious effects of malaria [16].

One of the most prevalent and cost-effective vector control strategies for malaria prevention is insecticide-treated bed nets [16–18]. It is treated with insecticidal chemicals and requires continued treatment, and ITNs are also effective in malaria prevention in high-endemic areas [3, 19]. As a result, the distribution and usage of ITNs are key interventions for malaria infection prevention control in underdeveloped countries, such as Ethiopia [16, 17].

The World Health Organization (WHO), through its 2015 Roll Back Malaria (RBM) initiatives, set ambitious targets to significantly reduce the global malaria burden. These goals included achieving a 75% reduction in malaria cases and minimizing malaria-related deaths, with a focus on ensuring universal coverage and proper utilization of insecticide-treated nets (ITNs) [20]. To support this aspect of malaria prevention, significant efforts and resources have been allocated by donors [21]. However, the proper utilization and coverage of insecticide-treated bed nets (ITNs) among priority and high-risk groups remain very low [22]. For instance, studies indicate that only 19% of these groups adequately use ITNs [23]. Several factors contribute to this low utilization, including discomfort, lack of experience in hanging nets, ineffective educational campaigns, and a scarcity of ITNs [24–26].

A study supported by the United States Agency for International Development (USAID), conducted through the Newmark Project in partnership with the Academy for Educational Development (AED), reported varying levels of ITN utilization the night before the survey. Findings revealed that 73% of households in the Oromia region and 60% in the Amhara region used ITNs [27]. Bibugn District, located in Northwest Ethiopia, has distinct topographical features, malaria transmission patterns, and socio-demographic characteristics that may uniquely influence the utilization of insecticide-treated bed nets (ITNs) [28]. Although several studies on ITN use have been conducted across Ethiopia, most have focused primarily on utilization rates, with limited attention given to the factors influencing usage, especially in context-specific settings like Bibugn. Therefore, this study aimed to assess ITN utilization and its associated factors among households in Bibugn District, providing locally relevant evidence to inform targeted malaria prevention strategies.

## Methods

### Study area and periods

The study was conducted in Bibugn district, North West Ethiopia; located 81 km from Debra Markos which is east Gojjam zone town 211 km from Bahirdar which is capital city of Amhara region and 381 km from Addis Ababa, the capital city of Ethiopia. According to 2007 census projection, The total population of the woreda was estimated to be 82,002, of which 40,190 are males and 41,812 are females [28].This study was conducted from January 30, up to March 30, 2024.

### Study design

Community based cross-sectional study design was used.

### Study population

All households living in the selected Kebeles of Bibugn district that owned at least one insecticide-treated net (ITN) were considered the study population. Households that had resided in the selected Kebeles for six months or more were included in the study. However, households were excluded if the respondent was severely or critically ill during the study period.

### Sample size calculation and sampling procedure

The sample size for the survey was calculated using the formula for a single population proportion, with assumptions of a 95% confidence interval, 5% margin of error, and an estimated 73% of households using ITNs based on a prior study in Ethiopia [29]. With 95% confidence interval, the marginal error of 5% (0.05), design effect (1.5) and non-response rate 10%, the resulting calculation for a total sample size was 500 households.

A two-stage sampling technique was employed. In the first stage, 10 kebeles (2 urban and 8 rural) were randomly selected from a total of 20. In the second stage, households were allocated proportionally across the selected kebeles based on a total of 1,489 registered households. Systematic random sampling was then applied, using a sampling interval of 3 (1489/500 ≈ 3). The first household was selected by lottery, and subsequent households were identified at regular intervals. For households where no eligible respondent was available, up to three revisits were conducted before being classified as non-respondents, confirmed through neighbors.

### Variables

*Dependent variables*: ITN utilization among Households.

*Independent variables*: Socio-demographic characteristics of Households (such as Age, educational status. Marital status, Religion, Occupation, monthly income, residence, and family size), Health information (Health worker, media), Personal factors such as (knowledge, practice), Housing condition such as (number of room, number of bed).

### Data collection tool

An interviewer-administered questionnaire was used to collect data. The questionnaire included sections on sociodemographic characteristics, knowledge, and health-related information relevant to insecticide-treated net (ITN) use. Eligible respondents were adult household members, preferably the household head or spouse, who had resided in the household for at least six months and were knowledgeable about household practices related to ITN ownership and use. These individuals were selected to ensure they could provide reliable and accurate information on behalf of the household.

Data collection was conducted by eight trained data collectors and supervised by two field supervisors. Prior to data collection, all personnel participated in a one-day training session covering the study objectives, interview techniques, ethical considerations including confidentiality, and procedures for obtaining informed consent. Data collectors were selected based on their language proficiency, willingness to participate, and demonstrated professionalism. Data were collected using a standardized questionnaire format. To ensure accuracy and reliability, trained interviewers administered the tool and completed each questionnaire based on direct responses from the eligible respondents.

### Data quality management

The questionnaire was developed in English, translated into Amharic, and back-translated to ensure accuracy and consistency. It was pretested on 5% of the sample in a non-selected area of Bibugn district to assess clarity, relevance, and flow. Data collectors and supervisors received one day of training on the study objectives, ethical considerations, and data collection procedures to ensure quality. During data collection, completed questionnaires were reviewed daily by data collectors, supervisors, and the principal investigator to check for completeness and internal consistency. After data entry, data cleaning was conducted to ensure accuracy and prepare the dataset for analysis.

### Data processing and analysis

Data were entered, cleaned, and coded using EpiData version 3.1 and then exported to SPSS version 25 for analysis. Descriptive statistics were presented in text, tables, and graphs. Binary logistic regression was used to identify factors associated with ITN utilization. Variables with a p-value of less than 0.25 in bivariable analysis were considered for inclusion in the multivariable logistic regression model. The 0.25 threshold was selected to avoid excluding potentially important variables too early, as recommended in epidemiologic literature to minimize residual confounding. Final associations were considered statistically significant at a p-value less than 0.05 with 95% confidence intervals. Model fitness was assessed using the Hosmer–Lemeshow goodness-of-fit test (*p* = 0.56), indicating adequate model fit. The maximum likelihood ratio test was also used to check model assumptions.

## Results

### Sociodemographic characteristics of the respondents

A total of 492 households participated in the study, yielding a response rate of 98.40%. Among the respondents, 280 (56.91%) were male. The mean age of respondents was 31.4 years (SD ± 5.77). Most respondents, 4 (89.02%), were married, and 393 (79.88%) resided in rural areas. Regarding religion, 402 (81.70%) identified as Orthodox Christians. Educationally, 199 (40.45%) of the respondents were illiterate, and the primary occupation was farming, with 292 (59.35%) engaged in agricultural activities. The majority of households (243, 49.39%) had a family size of 1–3 members. The mean monthly income of the respondents was 3,273.48 Ethiopian Birr, with an SD of ± 2,457.81. Most households had fewer than two rooms (386, 78.46%) and fewer than two beds (437, 88.82%) (Table 1).

**Table 1:** Socio-demographic characteristics of the respondents in Bibugn district, Northwest Ethiopia, 2024 (n = 492)

Characteristics	Categories	Frequency	(%)
Sex of the respondent	Male	280	56.91
	Female	212	43.09
Age group	18–24	87	17.68
	25–44	230	46.75
	45–64	169	34.35
	> 64	6	1.22
Residence	Urban	99	20.12
	Rural	393	79.88
Religion	Orthodox	402	81.70
	Muslim	74	15.04
	Protestant	16	2.90
Marital status	Married	438	89.02
	Single	27	5.48
	Divorced	19	3.86
	Widowed	8	1.64
Education status	Can’t read and write	199	40.45
	Can read and write (had not formal education)	95	19.30
	Elementary education	120	24.30
	Secondary education	48	9.75
	College and above	30	6.20
Occupation	Farmer	292	59.35
	House wife	89	18.09
	Merchant	34	6.91
	Government employee	54	10.98
	Daily labor	17	3.45
	Others	6	1.22
Family size	1–3	243	49.39
	4–5	219	44.51
	> 5	30	6.10
Monthly income	< 500	45	9.15
	500–1000	77	15.65
	> 1000	370	75.20
Number of rooms	< 2	386	78.46
	> 3	106	21.54
Number of beds	< 2	437	88.82
	> 3	55	11.18

### Magnitude of insecticide treated bed net utilization and distribution

In the study area, 489 (99.39%) households received ITNs, with 50.61% owning one. Health workers provided ITN information to 63.61% of respondents, and 301 (61.18%) households used ITNs the previous night. Properly mounted ITNs were observed in 299 (60.77%) households. Most ITNs (67.48%) were under three years old. While 214 (43.49%) households had two beds, 50.61% had only one ITN per bed (Table 2).

**Table 2:** ITN utilization and related condition in Bibugn district, Northwest Ethiopia, 2024

Characteristics	Categories	Frequency	Percent (%)
Received information about ITNs and its benefits	Yes	383	77.84
	No	109	22.16
From whom you received information	Media	59	11.99
	Health worker	313	63.61
	NGOs	9	1.83
	Others	3	0.41
Accesses to ITNs	Yes	489	99.39
	No	3	0.61
Households used ITN previous night	Yes	301	61.18
	No	191	38.82
ITN properly mounted over sleeping area	Yes	299	60.77
	No	193	39.23
ITN tear (hole)	Yes	88	17.88
	No	404	82.12
Number of beds or places of sleep	One	214	43.49
	Two	240	48.78
	Three & above	38	7.73
Number of bed nets observed in household	One	249	50.61
	Two	182	36.99
	Three & above	61	12.40
Number of beds (places) observed with bed nets	One	305	61.99
	Two	156	31.70
	Three & above	31	6.31
The age of ITNs present in the household	< 3 years	332	67.48
	3–5 years	157	31.91
	> 5 years	3	0.61

**Fig. 1:**
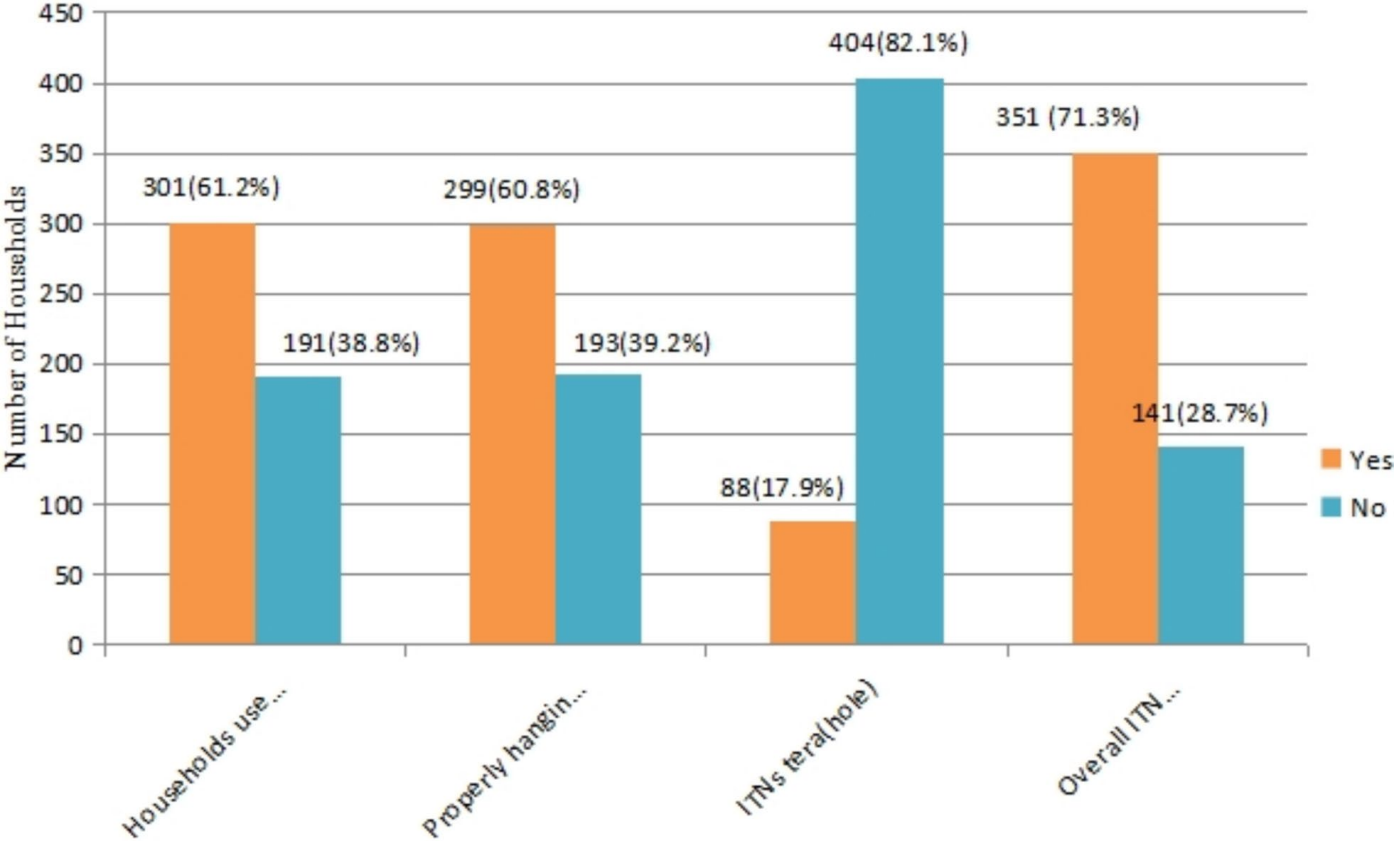
Proportion of ITNs utilization among Households in Bibugn District, North west Ethiopia, 2024

Utilization of ITNs was defined as the use of at least one insecticide-treated net by any household member on the night preceding the survey. Based on this definition, 351 (71.3%) households reported ITN utilization. This indicates that the majority of households effectively used ITNs, demonstrating a relatively high level of adherence to recommended malaria prevention practices (Fig. 1).

### Knowledge of Households about malaria and insecticide treated bed nets

About 473 (96.14%) respondents knew about malaria, and 318 (64.63%) of them identified mosquito bites as the cause of malaria transmission, while others mentioned alternative modes of transmission. Two hundred eighty-eight (58.54%) of the respondents were able to identify children under 5 years of age as the priority group for malaria prevention within households.

A total of 327 respondents (66.46%) reported using insecticide-treated nets (ITNs) as a priority measure for malaria prevention at the household level. Regarding malaria symptoms, chilliness was mentioned by 365 respondents (74.19%), fever by 256 (52.03%), headache by 137 (27.87%), vomiting by 45 (9.15%), and loss of appetite by 42 (7.80%). One respondent reported no knowledge of any symptoms.

Concerning respondents' knowledge about ITN prevention mechanisms, 293 (59.55%) reported that ITNs act as a physical barrier to prevent mosquito bites. Additionally, more than half of the respondents (70.12%) identified that ITNs should be used every night. The majority (90.04%) of respondents believed that ITN use does not pose any problems. Of the total participants, 34 respondents (7.2%) reported discomfort caused by heat when using ITNs (Table 3).

**Table 3:** Knowledge of households about malaria and insecticide treated bed net in Bibugn district, Northwest Ethiopia, 2024 (n = 492)

Characteristics	Categories	Frequency	Percent (%)
Know malaria disease	Yes	473	96.14
	No	19	3.86
Transmission of malaria	Mosquito bite	318	64.63
	By flies	21	4.27
	Due to water	27	5.49
	Not known	19	3.86
Priority given for malaria prevention within the household	< 5 children	288	58.54
	Pregnant women	257	52.23
	Adult	13	2.64
	Old age	15	3.05
Malaria prevention mechanism	Chemical spray	130	26.42
	Draining stagnant water	116	23.58
	Environment management	104	21.14
	ITN utilization	327	66.46
	Not known	17	3.45
Symptom of malaria	Fever	256	52.03
	Headache	137	27.85
	Chills	365	74.19
	Loss of appetite	42	8.53
	Vomiting	45	9.15
	Not known	9	1.83
ITN mechanism of action	Physical barriers	293	59.55
	Kills mosquito	167	33.49
	Not known	19	3.86
When to utilize ITN	Every night	345	70.12
	Sometimes	38	7.72
	Seasonally	87	17.68
	Not known	20	4.06
Any problem if ITN utilized	Yes	49	9.96
	No	443	90.04
List of problems if ITN utilized	No comfort	20	4.10
	Cause heat	34	7.20
	Air hanger	4	0.80
	Not known	2	0.40

Of the total participants, 269 (54.67%) respondents had good knowledge about ITNs and malaria, while 223 (45.33%) had poor knowledge. Among the respondents with good knowledge, 209 (59.54%) properly utilized ITNs. In contrast, among those with poor knowledge, 142 (40.46%) properly utilized ITNs (Supplementary file 1).

### Reasons for not using ITN by households

According to the response obtained from the respondents, old and worn out nets (12.81%), Lack of sufficient space to hang the net (8.73%), Suffocation/ too hot (4.24%), and not others (2.92%) were the reasons that were mentioned for not utilizing ITN (Supplementary file 2).

### Factors associated with ITN utilization among households

Bivariate analysis showed that variables such as sex, age, residence, educational status, monthly income, access to information about ITNs and their benefits, and knowledge were associated with ITN utilization (*p* ≤ 0.25). These variables were included in the multivariable analysis. In the multivariable logistic regression model, sex, access to information, monthly income, and educational status remained significantly associated with household ITN utilization (*p* < 0.05). The results of the multivariable logistic regression analysis indicated that women were 1.54 times less likely to use ITNs compared to men (AOR = 1.538; 95% CI 1.157, 3.135). This study also revealed that households with information about ITNs and their benefits had a 1.3 fold higher likelihood of utilizing ITNs compared to those without such information (AOR = 1.310; 95% CI 1.125, 3.229). Additionally, households earning more than 1,000 Ethiopian Birr in monthly income had 1.37 times higher odds of utilizing ITNs compared to those earning less than 500 Ethiopian Birr (AOR = 1.372; 95% CI 1.038, 3.184). Furthermore, the study found that households in which the respondant was unable to read and write had a 76.3% lower likelihood of ITN utilization compared to those with a college-level education or higher (AOR = 0.223; 95% CI 0.026, 0.871) (Table 4).

**Table 4:** Bivariable and multivariable analysis factors associated with ITN utilization in Bibugn district, Northwest Ethiopia, 2024 (n = 492)

Variables	ITN utilization				
	Yes	No	COR (95%CI)	AOR (95% CI)	*p* value
Sex of the respondent					
Male	213	67	1	1	**0.003**
Female	138	74	**1.704 (1.239, 3.613)**	**1.538 (1.157, 3.135)**	
Age group					
18–24	66	21	1.571 (0.263, 2.639)	0.618 (0.278, 1.998)	0.653
25–44	175	55	1.591 (1.104, 2.794)	0.762 (0.364, 1.873)	0.714
45–64	106	63	0.841 (0.396, 1.948)	0.857 (0.389, 1.887)	0.097
> 64	4	2	1	1	
Residence					
Urban	72	27	1.089 (0.673, 3.108)	1.179 (0.541, 2.798)	0.543
Rural	279	114	1	1	
Educational status					
Can’t read and write	127	72	**0.057 (0.014, 0.581)**	**0.237 (0.026, 0.871)**	**0.001**
Able to read and write	68	27	0.387 (0.127, 1.24)	0.583 (0.142, 1.891)	0.182
Elementary education	92	28	0.505 (0.132, 0.952)	0.633 (0.131, 1.362)	0.215
Secondary education	38	10	0.584 (0.128, 2.112)	0.785 (0.135, 1.993)	0.411
College and above	26	4	1	1	
Monthly income					
< 500	41	36	**0.342 (0.043, 0.649)**	**1.372 (1.038, 3.184)**	**0.002**
500–1000	26	19	0.414 (0.162, 1.342)	1.231 (0.594, 2.854)	0.274
> 1000	284	86	1	1	
Having information about ITNs and its benefits					
Yes	271	112	**0.877 (0.428, 0.994)**	**1.310 (1.125, 3.229)**	**0.000**
No	80	29	1	1	
Knowledge of the respondents					
Good	209	60	1.986 (1.1361, 3.145)	1.178 (0.642, 1.681)	0.543
Poor	142	81	1	1	

Bold values indicate statistically significant results, meaning the association is unlikely to be due to chance (p 0.05)

(*p* < 0.05) statistically significant

## Discussion

This study was designed to assess the prevalence and associated factors influencing insecticide-treated net (ITN) utilization among households in Bibugn District, Northwest Ethiopia. In addition to identifying the associated factors, the study also aimed to examine the actual utilization of ITNs among the households. The findings indicated that 71.3% of households used ITNs the night before the data collection day. This result is consistent with findings from Ilu Galan District in the Oromia Region of Ethiopia, where 72.2% of households reported ITN utilization [30], Arbaminch town in southern Ethiopia (71%) [29], Alamata district in northern Ethiopia (73%) [10], Harari regional state, Ethiopia (73.3%) [31], Burkina Faso (70%) [32], and Nigeria (75.4%) [32]. This may be because all of the above studies, including the present one, were conducted in malaria-endemic areas, where the risk of infection might have compelled households to use ITNs out of fear of contracting malaria.

However, the finding of this study is higher than that of the 2016 Ethiopian Demographic and Health Survey (EDHS), which reported that only 16.6% of households utilized ITNs [33], Northern Ethiopia (58.4%) [34], Sodo zuria woreda Southern Ethiopia (56.89%) [35], Mesekan District, Gurage Zone, Southern Ethiopia (58.2%) [36], Dawo district, Southwest Shoa Zone, Oromia, Ethiopia (55.5%) [37], Asgede Tsimbla District, Northern Ethiopia (63.1%), at a tertiary hospital in Nigeria 43.7% [4, 38]. This discrepancy may be attributed to differences in study settings, periods, and awareness levels. The higher utilization rate in the present study could be due to intensified malaria prevention campaigns, better access to health information, increased availability and distribution of ITNs in recent years, or heightened perceived risk of malaria in the study area [39].

The finding of this study is less than the result obtained from a study conducted among settlers in southwest Ethiopia [40] which found 80% of households used a bed net the night before the study, Afar (79.1%) [41], and WHO recommendation of ITN utilization to be in Ethiopia (80%) [42]. The disparity between this study and that of southwest Ethiopia study might be explained by the time gap between this study and the southwest Ethiopia study during which ITN distribution took place before the study was conducted. Others reason for the discrepancy of the 2 studies might be due to differences in the sociodemographic and socio-economic profiles of the study populations [43].

In this study, sex, educational status, monthly income, and having information about ITNs and their benefits were found to be statistically significant predictors of household ITN utilization. According to the findings of this study, being female was identified as a predictor of ITN utilization. This finding is consistent with a study conducted at Addis Zemen Hospital in Northern Ethiopia [44], Raya Alamata districts of Ethiopia [10], Ilu Galan District, Oromia Region, Ethiopia [30], and Arbaminch, Ethiopia [29] that found that females were less likely to utilize ITN than males. This might be also due to sociocultural background in which men are given priority over women in Ethiopia [45]. Moreover, this study was conducted in rural areas where males were more educated than females that lead males to have more awareness about ITN utilization [30]. This result is inconsistent with a study conducted in in Kersa Woreda, Jimma Zone, Southwest Ethiopia [46], in 7 sub-Saharan African countries [47] that showed females were identified as more likely utilized ITNs than males. The reason for this disparity might be due to difference in study setting and socio-cultural back ground of the communities.

Households in which the respondant was unable to read and write were significantly less likely to utilize ITNs compared to those with a college-level education or higher. This finding underscores the critical role of education in promoting health-protective behaviors. It is consistent with the results of a study conducted at Addis Zemen Hospital in Northwest Ethiopia, which also highlighted lower ITN utilization among individuals with limited or no formal education [48] and Damot pulasa district, Southern Ethiopia [49]. This difference might be due to educated Households may know the consequences of malaria if they did not use ITNs. Another possible explanation might be due to the fact that educated population can easily read and understand the information regarding malaria prevention mechanisms including ITNs.

In this study, earning more than 1,000 Ethiopian Birr per month was positively associated with ITN utilization. Households with higher monthly income were more likely to use ITNs compared to those with lower income, possibly due to better access to health information, improved living conditions, and increased health awareness. This finding is consistent with a study conducted in Southwest Ethiopia, which also reported a positive relationship between household income and the utilization of ITNs [50] and Ilu Galan District, Oromia Region, Ethiopia [30] where households with higher wealth indexes were more likely to utilize ITN. This is because those households that have better income can afford ITNs and utilize than those with low monthly income. This result is inconsistent with a study conducted in Ghana [51]. This could be due to the reason that rich families may consider other prevention methods and this might have affected the LLIN utilization [52].

In this study, households that had received information about ITNs and their benefits were 1.3 times more likely to utilize ITNs compared to their counterparts. Access to information likely improved awareness of the importance of ITNs in preventing malaria, which in turn promoted their usage. This finding is consistent with a study conducted in Fogera, Northwest Ethiopia, where exposure to health education was also found to significantly influence ITN utilization [53], Mihrab Abaya Gamo Gofa [54], Gambelia [44], Awabel District [55], and Jawi Northwest Ethiopia [56]. This could be attributed to the fact that Households who had received health education about insecticide treated bed nets were better aware of the importance ITNs to prevent malaria [57].

Efforts to increase ITN use should ensure that education and awareness activities are inclusive and accessible to underserved groups, including low-literacy households, poorer families, and men. Messages must use local languages and culturally relevant approaches that reflect community realities. Segmenting audiences, monitoring progress, and adapting communication strategies are crucial for effective and sustainable behavior change. Addressing social and gender norms will further enhance the equity and impact of awareness efforts.

### Strengths and limitation of study

A key strength of this study was the use of direct observation to validate ITN utilization, enhancing data accuracy. However, this study had some limitations. The use of logistic regression may have overestimated associations for common outcomes, as alternative models like modified Poisson regression were not feasible. Self-reporting and recall bias might also have affected responses. Moreover, the cross-sectional design limits causal inference and generalizability to all malaria-endemic areas.

## Conclusion

In this study, the utilization of insecticide-treated bed nets (ITNs) among households was 71.3%, below the WHO recommendation of at least 80% [58] household coverage and use for effective malaria prevention. Sex, educational status, monthly income, and access to information about ITNs and their benefits were identified as significant predictors of utilization. Specifically, being male, having a college-level education or higher, earning a higher monthly income, and receiving health information were positively associated with ITN use. To achieve and maintain the WHO-recommended coverage, interventions must go beyond traditional education campaigns to incorporate inclusive, gender-responsive, and literacy-sensitive communication strategies. Community engagement, audience segmentation, and continuous evaluation of education programs are vital to ensure that interventions effectively reach all segments of the population and reduce the malaria burden.

## Supplementary Information

Additional file1 (DOCX 39 KB)

## Data Availability

The datasets analysed during the current study are available from the corresponding author upon reasonable request.
