# Supplementary material for: Utilization of insecticide-treated bed nets and key factors among households in resource-limited settings, Northwest Ethiopia
Source: Malar J. 2025 Nov 28;25:16. doi: 10.1186/s12936-025-05698-8 (PMC12784607; doi:10.1186/s12936-025-05698-8)
Supplement: Supplementary file 1 — Additional file1 (DOCX 39 KB) [file 12936_2025_5698_MOESM1_ESM.docx]

Supplementary file 1. The distribution of ITN utilization on knowledge status towards ITNs utilization nets in Bibugn District, Northwest Ethiopia, 2024.

|  | | LLINs utilization | | | |
| --- | --- | --- | --- | --- | --- |
|  |  | Properly Utilized | Not properly Utilized | | Total |
| Knowledge about ITNs Utilization | Good | 209(59.54%) | 60(42.55%) | 269(54.67%) | |
|  | Poor | 142(40.46%) | 81(57.45%) | 223(45.33%) | |

Supplementary file 2: Reasons for not utilizing ITNs the night before data collection day in Bibugn district, Northwest Ethiopia, 2024 (n = 141).
